# Professor Haeckel’s Life in Ceylon

**Published:** 1882-12

**Authors:** 


### PROFESSOR HAECKEL'S LIFE IN CEYLON.

My great resource as an article of diet was the fruit which abounded at every meal and made up for all that I suffered from Babua's curries. Next to the bananas of every variety, of which I consumed several at every meal, my standing dessert consisted of mangoes (*Mangifera indica*), egg-shaped green fruit, from three to six inches long; their cream-like golden pulp has a faint but distinct aroma of turpentine. The fruit of the passion-flower (*passiflora*) was very pleasant to my taste, reminding me of the gooseberry. I was less pleased with the renowned custard-apple, the scaly fruit of the *Annona squamosa*, and with the Indian almond, the hard nut of the *Terminalia catappa*. There are singularly few apples and oranges in Ceylon; the latter remain green, and are sour and not juicy ; but the want of cultivation is doubtless chiefly answerable for the inferiority of this and other fruits; the Singhalese are far too easy going to make any progress in horticulture. Refreshed with my modest repast, I employed the hot hours of mid-day—from twelve to four o'clock—in anatomical or microscopic work in making observations and drawings, and in the preservation and storing of my collected objects. The evening hours, from four to six o'clock, were generally occupied with some lovely country excursion; sometimes I made a water color sketch, sometimes I sought to perpetuate one of the beautiful views in photography. Now and then I shot apes and birds in the woods, or collected insects and snails, or hunted among the coral reefs on the shore, adding many curious objects to my collection. Richly laden, I returned to the Rest House an hour or less before sunset, and worked for another hour at the preservation and arrangement of my specimens. At eight o'clock my second chief meal, or dinner, was served. The *pièce de résistance* at this was again the inevitable curry and rice, followed sometimes by a fish or a crab, which I enjoyed immensely, and then by some dish composed of eggs or meal, and finishing again with delicious fruit. * * * The important question of "what to drink" seemed likely at first to prove a difficult one. The ordinary drinking water of the lowlands of Ceylon is considered very bad and unwholesome, the highlands, on the contrary, being rich in springs of the purest and freshest water. The great rains which fall daily on the island bring down a mass of mineral and vegetable deposit into the rivers, and the stagnant water of the lagoons is not unfrequently in communication with them. It is not customary to drink the water unless boiled or made into tea, or with the addition of claret or whiskey. My friend

Scott had given me an abundant supply of the last-named beverage but on the whole I found no drink so pleasant and refreshing, as well as wholesome, as the fresh milk of the cocoa-nut.

My frugal dinner at an end, I usually took a solitary walk on the shore, or delighted my eyes with the sight of the illumination of the palm woods by myriads of fire-flies and glow-worms. Then I made a few entries in my note-book, or tried to read by the light of a cocoa-nut oil-lamp. But I was generally quite tired enough to go to bed soon after 9 o'clock, after another careful shaking of the clothes for the expulsion of scorpions and millipeds.

The great black scorpion (nearly a foot long) is so common in Ceylon that I once collected half a dozen in the course of an hour. Snakes exist also in great numbers. Slender green tree-snakes hang from almost every bough, and at night the great rat-snake (*Coryphodon Blumenbachii*) hunts rats and mice over the roofs of the huts. Although they are harmless and their bite not poisonous, it is by no means a pleasant surprise when one of these rat-snakes, five feet long, suddenly drops through a hole in the roof into one's room, occasionally alighting on the bed.

On the whole, however, my nights in Belligam were but little disturbed by animal intruders, although I was often kept awake by the howling of jackals and the uncanny cry of the devil-bird (a kind of owl, *Syrnium Indrani*) and other night birds. The bell-like cry of the pretty little tree frogs, which make their dwellings in the cups of large flowers, acted rather as a slumber song. But I was far oftener kept awake by the whirl of my own thoughts, by the recollection of the many events of the past day, and the anticipation of that which was to come. A brilliant succession of lovely scenes, of interesting observations and varied experiences mingled in my brain with plans of fresh enterprise and new discoveries for the morrow. —*Deutsche Rundschau.*

